# LiteFreqMamba:lightweight frequency-enhanced Mamba for efficient 3D brain tumor segmentation

**DOI:** 10.3389/fonc.2026.1928589

**Published:** 2026-09-04

**Authors:** Xiangning Hou, Jun Yao, Qiaochu Li, Caocao Xu, Wenxin Dong

**Affiliations:** The Engineering & Technical College of Chengdu University of Technology, Leshan, China

**Keywords:** brain tumor segmentation, frequency enhancement, lightweight, Mamba, state space models

## Abstract

**Introduction:**

State Space Models (SSMs) have demonstrated strong potential for 3D brain tumor segmentation owing to their linear computational complexity. However, conventional Mamba-based models are often limited by spectral bias, which favors low-frequency information while overlooking high-frequency boundary details, as well as by spatial disruption introduced by 1D scanning.

**Methods:**

We propose LiteFreqMamba, a novel frequency-enhanced architecture for accurate and efficient 3D brain tumor segmentation. LiteFreqMamba is designed to improve boundary representation and spatial modeling through several key components. First, a Decomposed Frequency-Spatial Convolution (DFS-Conv) encoder is introduced to explicitly decompose features and capture high-frequency boundary information in shallow layers, thereby alleviating boundary ambiguity. Second, a Mamba-Attention Hybrid Bottleneck (MAHB) is developed to preserve spatial structure by combining the 2D-Selective-Scan (SS2D) mechanism for linear-complexity spatial mixing with dense self-attention for finegrained pixel-wise dependency modeling. In addition, Frequency-Calibrated Skip Connections (FCSC) are proposed to dynamically suppress noise in high-frequency feature injection, and a Lightweight 3D Convolutional (LWT-3D Conv) decoder is employed for efficient feature reconstruction.

**Results:**

Extensive experiments on the BraTS2020 and BraTS2021 benchmarks show that LiteFreqMamba surpasses existing efficient segmentation models and achieves a better balance between inference speed and segmentation accuracy.

**Discussion:**

LiteFreqMamba is designed to improve high-frequency boundary representation and preserve spatial dependencies in Mamba-based architectures. The proposed framework provides an efficient and accurate solution for 3D brain tumor image segmentation.

## 1 Introduction

Gliomas are among the most aggressive and heterogeneous primary brain tumors, characterized by complex sub-regions and infiltrative boundaries (1–3). Accurate and automatic segmentation of these substructures from multi-modal Magnetic Resonance Imaging (MRI) is a prerequisite for quantitative diagnosis, treatment planning, and postoperative monitoring (4). Since the inception of the Brain Tumor Segmentation (BraTS) challenge (5), deep learning methodologies have revolutionized this field, pushing the boundaries of segmentation accuracy.

Convolutional Neural Networks (CNNs), particularly the Fully Convolutional Network (FCN) (6) and U-Net variants (7–9), have long served as the de facto standard for volumetric medical segmentation. The encoder-decoder structure with skip connections effectively recovers spatial resolution, while operations like dilated convolutions (10) enlarge the receptive field. Despite their dominance, CNNs suffer from intrinsic limitations in modeling long-range dependencies. The local inductive bias of convolution operations restricts the effective receptive field to a small neighborhood, preventing the network from fully capturing the global shape and contextual information of large or multi-focal tumors (11, 12). Although stacking more layers can theoretically increase the receptive field, it often leads to optimization difficulties and diminishing returns.

To surmount the limitations of CNNs, Vision Transformers (ViTs) (13) have been introduced to the medical imaging domain, leveraging Self-Attention (SA) mechanisms to model global interactions (14, 15). Models like SwinUNETR (11) and UNETR (14) have demonstrated superior performance by treating volumetric data as sequences of tokens. However, this superior global modeling capability comes at a steep price: the computational complexity of self-attention grows quadratically with the number of tokens (16). For high-resolution 3D volumetric data, the number of tokens increases cubically, leading to prohibitive memory consumption and latency. Consequently, existing Transformer-based methods often resort to aggressive patch embedding or window-based attention (17), which may disrupt the continuity of anatomical structures and compromise fine-grained details (18).

Recently, Structured State Space Models (SSMs), particularly the Mamba architecture (19), have emerged as a formidable competitor to Transformers. By introducing a data-dependent selection mechanism into continuous-time state-space models, Mamba achieves global receptive fields with linear computational complexity (20–22). Initial adaptations of Mamba in medical segmentation, such as U-Mamba (23) and SegMamba (24), have shown promising results in processing long sequences of 3D data efficiently. However, applying standard 1D Mamba directly to 3D medical images presents two critical challenges. First, flattening volumetric data into 1D sequences disrupts the inherent spatial structure and introduces direction sensitivity. Second, and more importantly, standard SSMs have been associated with a tendency to emphasize low-frequency structural information (25), which may lead to insufficient representation of high-frequency boundary details (26). This potential limitation is particularly relevant to glioma segmentation, where tumor margins are often diffuse, irregular, and difficult to distinguish from surrounding tissue.

To address these problems, we propose LiteFreqMamba, a lightweight frequency-enhanced framework for 3D brain tumor segmentation. Its core objective is to preserve the efficiency of state-space modeling while improving spatial dependency modeling and high-frequency boundary representation. Specifically, the proposed Decomposed Frequency-Spatial Convolution (DFS-Conv) encoder applies (2 + 1)D wavelet-based decomposition in shallow stages to preserve boundary cues. The Mamba-Attention Hybrid Bottleneck (MAHB) combines the Visual State Space (VSS) block with the 2D-Selective-Scan (SS2D) mechanism and self-attention to integrate efficient global modeling with dense feature interaction. The Frequency-Calibrated Skip Connections (FCSC) use Squeeze-and-Excitation (SE) based channel calibration to suppress task-irrelevant high-frequency noise during feature fusion. A lightweight 3D convolutional decoder is used for efficient feature reconstruction.

The main contributions are as follows:

1. We propose LiteFreqMamba, a lightweight frequency-spatial architecture designed to improve high-frequency boundary representation and spatial dependency modeling in Mamba-based 3D segmentation.
2. We develop DFS-Conv and MAHB to preserve high-frequency boundary cues while combining efficient global modeling with local feature interactions.
3. We introduce FCSC to dynamically recalibrate high-frequency skip features and improve encoder–decoder fusion.
4. Experiments on BraTS2020 and BraTS2021 show competitive segmentation performance with 4.96M parameters and 123.98 GFLOPs. The model uses 82.61% fewer parameters than SegMamba and 92.03% fewer than SwinUNETR.

The methodological contribution of LiteFreqMamba does not rely on introducing a new wavelet transform, state-space operator, or attention mechanism in isolation. Instead, it addresses the joint problem of boundary preservation, spatial dependency modeling, and computational efficiency through a coordinated architecture. Specifically, frequency decomposition is performed in shallow encoder stages to preserve boundary-related high-frequency cues, SS2D-based state-space modeling and self-attention are combined at the low-resolution bottleneck to capture complementary global and dense dependencies, and frequency-calibrated skip connections selectively regulate the transmission of high-frequency features. This task-oriented coordination distinguishes LiteFreqMamba from methods that employ frequency enhancement, Mamba-based modeling, or attention-guided fusion as isolated components.

## 2 Related work

### 2.1 CNNs and transformers in medical segmentation

For the past decade, Convolutional Neural Networks have served as the cornerstone of medical image analysis. The encoder-decoder architecture, popularized by U-Net (8), facilitates the capture of multi-scale features. Several 3D variants, such as V-Net (12) and 3D U-Net (7), extended this paradigm to volumetric data. More recently, nnU-Net (27, 28) demonstrated that a well-configured U-Net can achieve state-of-the-art performance without complex architectural changes. However, convolution operations possess a limited effective receptive field due to their local inductive bias, making it difficult to model long-range dependencies essential for segmenting large or multi-focal tumors (11).

To address the limitations of CNNs, ViTs (13) introduced the self-attention mechanism to computer vision. TransBTS (15) was among the first to utilize a Transformer bot-tleneck to capture global features in 3D MRI scans. Subsequently, hierarchical Trans-formers like Swin Transformer (17) were adapted into segmentation frameworks, yielding models such as SwinUNETR (11) and nnFormer. While these methods significantly improve performance, the computational complexity of standard self-attention is quad-ratic with respect to the number of tokens. For high-resolution 3D images, this results in prohibitive memory costs (29). Although window-based attention mitigates this to some extent, it limits the global context to local windows, partially defeating the purpose of using Transformers (18).

### 2.2 State space models and vision Mamba

Structured State Space Models have recently gained traction as a powerful alternative for long-sequence modeling. Originating from classical control theory and the HiPPO framework (30), modern SSMs like S4 (20) map 1D sequences through implicit latent states. Gu et al. further introduced Mamba (19), which incorporates a selective scan mechanism S6 to achieve input-dependent weighting, enabling linear-time complexity with performance comparable to Transformers.

However, standard Mamba models process data sequentially, which is suboptimal for 2D/3D visual data where spatial locality is crucial. To address this, Vim (Vision Mamba) (31) emerges as an new vision backbone with bidirectional state-space models; VMamba (32) introduced the SS2D, traversing the image in multiple directions to model 2D dependencies without breaking spatial structure. In the medical domain; U-Mamba (23) and SegMamba (24) have adapted these blocks for volumetric segmentation. Despite their efficiency, these methods primarily focus on spatial modeling and often overlook the inherent spectral bias of SSMs (25), which tends to prioritize low-frequency components. Our work differs by integrating (2 + 1) D frequency-domain learning to explicitly compensate for the loss of high-frequency boundary details in Mamba-based backbones.

### 2.3 Frequency-aware deep learning

While most deep learning models operate in the spatial domain, exploring the frequency domain offers a complementary perspective. Early works utilized the Fourier Transform (FFT) to perform global spectral filtering (33), such as FcaNet (34). However, FFT lacks spatial localization, making it less effective for local boundary delineation.

Wavelet Transform (WT), which provides simultaneous time-frequency localization, offers a better solution for dense prediction tasks. WaveViT (35) and Multi-level Wave-let-CNN (36) utilize Discrete Wavelet Transform (DWT) to enable lossless downsampling and texture enhancement. In 3D medical imaging, Dual-Domain Learning (37) has been proposed to leverage K-space data. However, applying 3D DWT directly often leads to channel explosion. Our proposed (2 + 1) D DFS-Conv addresses this by factorizing the volumetric frequency analysis into spatial 2D DWT and depth-wise 1D convolution, efficiently capturing high-frequency edge cues while maintaining computational feasibility.

### 2.4 Feature fusion and skip connections

Skip connections are a hallmark of U-Net-like architectures, facilitating the recovery of spatial details by transferring encoder features to the decoder (8). Standard implementations typically employ simple channel concatenation or element-wise addition. However, this naive fusion often suffers from the semantic gap between low-level encoder features and high-level decoder features (9). To address this, advanced mechanisms such as Attention U-Net (38) introduced attention gates to spatially suppress background regions, while UNet++ (9) utilized nested dense pathways to reduce the semantic dissimilarity. Despite these advancements, most existing fusion strategies focus primarily on spatial attention, often overlooking the channel-wise distribution of noise and signal. Unlike these spatial-centric approaches, this work explores a frequency-calibrated strategy. We aim to leverage channel attention mechanisms, such as Squeeze-and-Excitation (39), to dynamically filter out task-irrelevant high-frequency noise inherent in shallow features, thereby bridging the semantic gap more effectively for heterogeneous tumor segmentation.

## 3 Methodology

### 3.1 Overview of the proposed model

**Figure 1:**
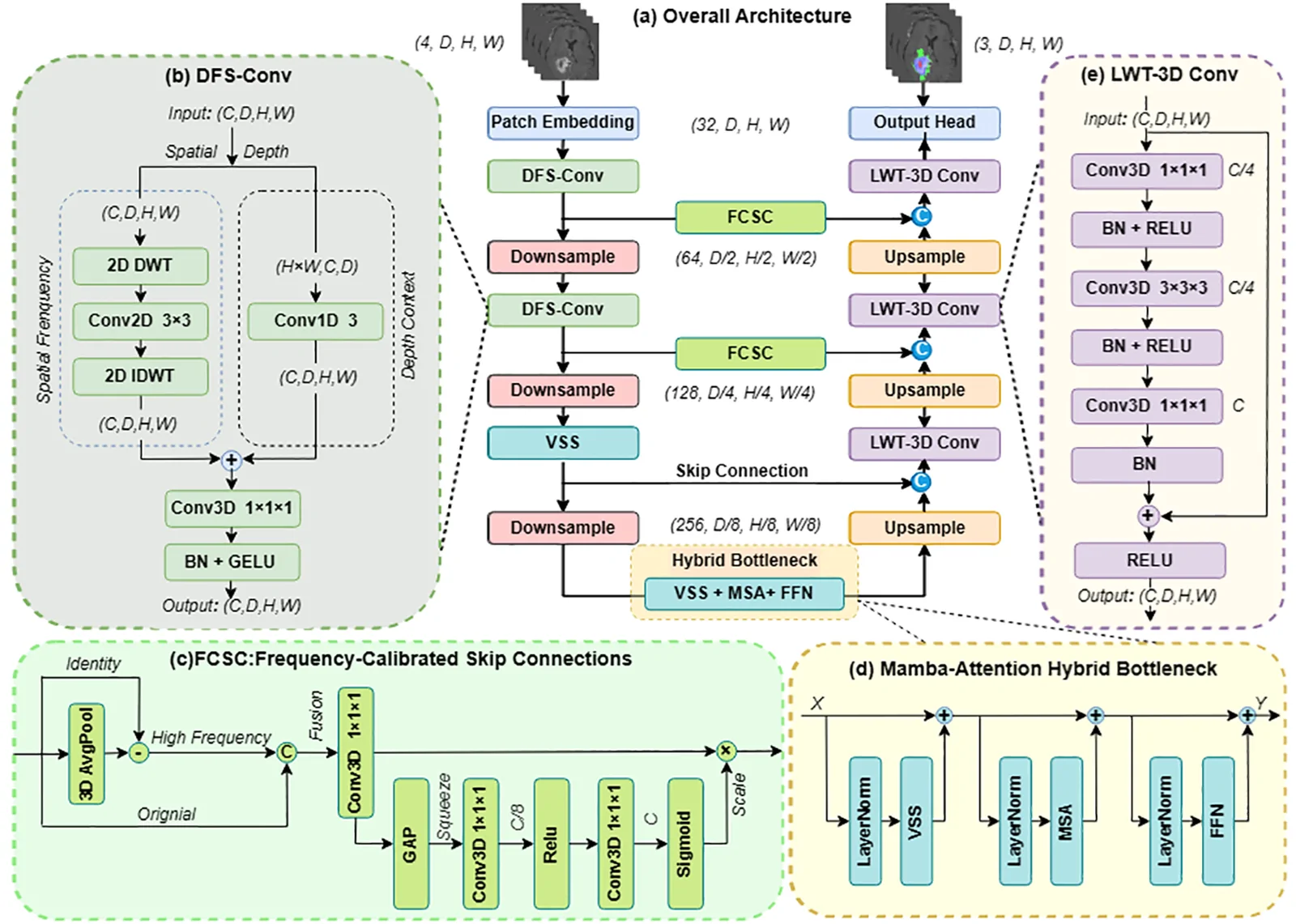
The LiteFreqMamba architecture. **(a)** Overall Architecture: The network adopts a hybrid U-shaped design with a Frequency-Aware Encoder and a Lightweight Decoder. **(b)** DFS-Conv: Used in shallow encoder stages to explicitly extract high-frequency boundary cues via (2 + 1) D wavelet decomposition. **(c)** FCSC: Dynamically filters and injects high-frequency details from the encoder to the decoder using SE-based calibration. **(d)**MAHB: Mamba-Attention Hybrid Bottleneck: Sequentially integrates Mamba and Self-Attention to capture dual-view context. **(e)** LWT-3D Conv: Employed in the decoder for efficient feature reconstruction with reduced parameters.

The proposed LiteFreqMamba utilizes a hierarchical encoder-decoder architecture tailored for efficient volumetric segmentation, as illustrated in **Figure 1a**. The encoder is composed of four stages designed to extract multi-scale frequency-aware features. Notably, the shallow stages uniquely incorporate DFS-Conv blocks. Unlike standard downsampling, DFS-Conv employs a (2 + 1) D discrete wavelet transform to explicitly decompose input volumes into spatial frequency sub-bands, effectively capturing high-frequency boundary cues while maintaining depth consistency. In the deep stages, the network transitions to VSS blocks, leveraging 2D-Selective-Scan mechanisms to model long-range global dependencies with linear complexity. The bottleneck further integrates a Mamba-Attention Hybrid module, synergizing the efficiency of SSMs with the dense correlation of Self-Attention. The decoder mirrors this hierarchy using Lightweight 3D Convolutional blocks to restore spatial resolution. Crucially, Frequency-Calibrated Skip Connections (FCSC) bridge the encoder and decoder, dynamically filtering task-irrelevant noise via channel calibration to ensure precise boundary reconstruction in the final prediction.

Given a 3D MRI input $\;X\in {\mathbb{R}}^{\text{C}\times D\times H\times W}$, the model progressively downsamples features through the encoder, refines them at the bottleneck, and upsamples them through the decoder to predict a voxel-wise segmentation map $\;Y\in {\mathbb{R}}^{N_{cls}\times D\times H\times W}$.

### 3.2 Frequency-aware encoder

The shallow layers of the encoder are responsible for extracting low-level features such as edges and textures, which are critical for precise boundary delineation. Standard 3D convolutions effectively capture local features but often neglect high-frequency boundary signals due to the smoothing effect of downsampling operations and inherent spectral bias (25). To mitigate this, we propose the DFS-Conv Block for the shallow layers. DFS-Conv Applying 3D Discrete Wavelet Transform (DWT) directly leads to an 8× channel explosion, which is memory-prohibitive for high-resolution volumetric data. We instead adopt a factorized strategy, as shown in **Figure 1b**. We treat the spatial (*H, W*) and depth (*D*) dimensions separately to achieve efficient frequency decomposition.

Formally, for an input feature *X*, we first reshape it into a sequence of 2D slices. Then, we perform 2D DWT on each slice using Haar wavelets, decomposing it into four frequency sub-bands: low-frequency approximation *X_LL_*​ and high-frequency details *X_LH_*, *X_HL_*, *X_HH_*​. This increases the channel dimension by 4× but reduces the spatial resolution by 2×, where $\;X_{spatial}\in {\mathbb{R}}^{4\text{C}\times D\times H/2\times W/2}$. These sub-bands are concatenated along the channel dimension, as defined in Equations 1, 2.

(1) $$X_{LL},X_{LH},X_{HL},X_{HH}=DWT2D(X)$$

(2) $$X_{freq}=DWT2D(X)=Concat[X_{LL},X_{LH},X_{HL},X_{HH}]$$

We then apply a 2D 3×3 convolution in the wavelet domain to refine these frequency features, followed by an Inverse DWT (IDWT) to reconstruct the spatial structure (Equation 3):

(3) $$Y_{spatial}=IDWT2D(Conv2D(X_{freq}))$$

Simultaneously, to ensure inter-slice continuity and capture volumetric context, we apply a lightweight 1D depth-wise convolution along the D dimension with a kernel size of 3 (Equation 4):

(4) $$X_{depth}=Conv1D(X)$$

The final output is the element-wise fusion of these two paths, followed by a 1×1×1 convolution for channel mixing (Equation 5):

(5) $$Y_{out}=Conv3D_{1\times 1\times 1}(Concat[X\_spatial,X\_depth])$$

This (2 + 1) D decomposition forces the network to attend to high-frequency components explicitly in the spatial domain while maintaining depth consistency, providing robust boundary cues for subsequent layers without incurring excessive computational cost.

### 3.3 Mamba-attention hybrid bottleneck

The bottleneck layer represents the most abstract semantic level of the network, where feature resolution is low but the receptive field requirement is maximal. While standard State Space Models like Mamba (19) excel at global sequence modeling with linear complexity, they typically flatten volumetric data into 1D sequences. This flattening process disrupts the inherent spatial structure of 3D anatomical features and introduces direction sensitivity—i.e., the model captures dependencies primarily in the scanning direction while neglecting neighbors in other directions. To address this, we propose a MAHB module, which synergizes the spatial awareness of VSS models with the dense correlation capability of Transformers. The module comprises three sequential residual stages: Visual State Space Mixing, Dense Spatial Correlation, and Channel Refinement. , as illustrated in **Figure 1d**.

Visual State Space Mixing: Instead of standard 1D scanning, we adopt the SS2D mechanism proposed in VMamba (32). This mechanism bridges the gap between 1D array scanning and 2D/3D visual structure. Specifically, the input feature map *X* is scanned along four distinct directions (top-left, bottom-right, top-right, bottom-left) to generate four independent sequences. Each sequence is processed by a distinct SSM to extract direction-aware features, which are then merged back to restore the original spatial structure. This allows each voxel to integrate information from all other voxels in a spatially continuous manner with *O(N)* complexity. Formally, the output of the VSS stage *X_vss_*​ is computed as Equation 6:

(6) $$X^{\prime }=VSS(LN(X))+X$$

where *VSS* denotes the Cross-Scan Module (CSM) that aggregates features from the four scanning directions, and *LN* represents Layer Normalization.

Dense Spatial Correlation: While *VSS* efficiently captures global context via scanning, it may still lack the pixel-perfect precision of direct “all-to-all” interactions, which are crucial for delineating complex tumor boundaries. To compensate for this, we append a Multi-Head Self-Attention module (16). Given the reduced resolution at the bottleneck, the quadratic complexity *O(N^2^)* of *MSA* is computationally negligible. This stage refines the *VSS* features by explicitly modeling the dense correlation matrix between every pair of voxels (Equation 7):

(7) $$X^{\prime \prime }=MSA(LN(X^{\prime }))+X^{\prime }$$

Channel Refinement: Finally, to enhance feature distinctiveness and facilitate channel interaction, we employ a Feed-Forward Network (FFN) with an inverted bottleneck structure and GELU activation. This stage performs point-wise non-linear transformation (Equation 8):

(8) $$Y=FFN(LN(X^{\prime \prime }))+X^{\prime \prime }$$

By sequentially integrating *VSS* and *MSA*, our hybrid bottleneck achieves Dual-view Context Aggregation, leveraging the linear efficiency of SSMs for global scope and the dense attention of Transformers for local precision, ensuring robust representation of heterogeneous tumor sub-regions.

### 3.4 Frequency-calibrated skip connections

In U-Net architectures, skip connections are pivotal for recovering fine-grained details lost during downsampling (8). However, a direct concatenation of encoder and decoder features often introduces a semantic gap (9, 27). The encoder features, especially those from our DFS-Conv layers, are rich in high-frequency details but also contain task-irrelevant noise. The decoder features, conversely, are semantically strong but spatially coarse.

To address this misalignment, we propose the FCSC module, as illustrated in **Figure 1c**. This module operates as a dynamic filter, selectively enhancing informative boundary cues while suppressing noise before fusion. The process consists of three sequential steps:

High-Frequency Injection: We first extract the high-frequency residual *X_HF_* by subtracting the low-pass filtered signal from the original encoder feature *X_enc_*. The low-pass signal is approximated via local average pooling (Equation 9):

(9) $$X_{HF}=X_{enc}-\;\text{AvgPool}(X_{enc})$$

This explicit extraction ensures that edge information is prioritized over smooth background variations.

Feature Fusion: We then concatenate the original encoder feature *X_enc_* with the extracted high-frequency residual *X_HF_* along the channel dimension. This creates a fused representation that contains both semantic context and sharp boundary details (Equation 10):

(10) $$X_{fused}=Conv3D_{1\times 1\times 1}(\text{Concat}[X_{enc},X_{HF}])$$

A 1×1×1 convolution is applied to project the concatenated features back to the original channel dimension C.

Dynamic Calibration: Finally, to filter out noise from the injected high-frequency components, we employ a Squeeze-and-Excitation block (39) for channel-wise recalibration. The SE block learns a set of channel weights $w\in {\mathbb{R}}^{\text{C}}$ based on global context, amplifying informative channels and suppressing noisy ones, as shown in Equations 11, 12:

(11) $$w=\sigma (W_{2}\delta (W_{1}GAP(X_{fused})))$$

(12) $$Y_{skip}=w⊙X_{fused}$$

where *GAP* denotes Global Average Pooling, *δ* is ReLU activation, and *σ* is the Sigmoid function, and ⊙ represents channel-wise multiplication. The calibrated feature map *Y_skip_* is then passed to the decoder for concatenation with the upsampled features.

### 3.5 Lightweight decoder

The decoder is responsible for reconstructing the high-resolution segmentation map from the encoded semantic features. Given that the feature maps in the decoder have high spatial resolution, applying standard 3D convolutions would result in excessive computational cost and memory usage. To address this, we design a Lightweight 3D Convolutional (LWT-3D Conv) Block, as depicted in **Figure 1e**. Instead of standard residual blocks, we adopt a bottleneck design to minimize parameters. Specifically, for an input feature with C channels, the block operates in three steps:

- Squeeze: A 1×1×1 convolution reduces the channel dimension to C/4, minimizing the computational burden for the subsequent spatial operation. The C/4 setting was selected as a compromise between reconstruction capacity and computational efficiency.
- Process: A 3×3×3 convolution performs spatial feature extraction in this low-dimensional space.
- Expand: Another 1×1×1 convolution restores the channel dimension to C.

This Squeeze-and-Expand strategy reduces the parameter count of the decoder, ensuring that LiteFreqMamba remains efficient even during the high-resolution reconstruction phase.

## 4 Results and discussion

### 4.1 Datasets and data preprocessing

In this work, all experiments are conducted on the BraTS2020 (2) and BraTS2021 (1) dataset. Only the publicly annotated training cohort is utilized, no official challenge validation and test data are used for local quantitative evaluation. We split the patient-wise cases into training, validation, and internal test set following an 8:1:1 ratio with a fixed random seed 42. To assess training variability, each method was trained independently using five random seeds {42, 123, 456, 789, 1024}. Four modalities (T1, T1ce, T2, FLAIR) are adopted as input, and tumor sub-regions including enhancing tumor (ET), peritumoral edema (ED), necrotic and non-enhancing tumor (NCR/NET) are evaluated. Following the official evaluation protocol, we merge these labels into three nested sub-regions: Whole Tumor (WT), Tumor Core (TC), and ET.

To mitigate overfitting and enhance the model’s generalization capability across diverse scanning protocols, we implement a comprehensive data processing pipeline using the MONAI framework (40). All multi-modal MRI scans are first co-registered, skull-stripped, and resampled to 1×1×1 mm^3^ isotropic resolution. We apply z-score normalization to each modality independently to standardize intensity distributions. During training, we employ rigorous on-the-fly augmentations. Spatial transformations include random 3D cropping to a patch size of 128×128×128, random affine rotations (± 30°), axis flipping, and elastic deformations to simulate anatomical variability. Intensity-wise, we apply random Gaussian noise injection, Gaussian smoothing, and gamma correction to mimic scanner-induced artifacts and contrast variations. These augmentations ensure robustness against the heterogeneity of tumor appearances.

### 4.2 Experimental setup

The proposed LiteFreqMamba is implemented in PyTorch 2.0.1 with CUDA 11.8 and MONAI 1.3.0. All experiments are conducted on a single NVIDIA RTX 4090 GPU. To ensure the fairness of the comparison, all the comparison models were re-trained using the same patient-level division, preprocessing procedure, input image size, data augmentation, optimizer, learning rate schedule, number of training iterations, loss function, and sliding window inference protocol as LiteFreqMamba. We employ the AdamW optimizer (41) with an initial learning rate of 1×10–^4^ and a weight decay of 1×10^-5^. A cosine annealing scheduler is used to decay the learning rate. The batch size is set to 2 to maximize GPU utilization. To ensure convergence, models are trained for 1000 epochs. We use a sliding window inference strategy with a window size of 128×128×128 and an overlap of 0.5 during testing to improve robustness at the patch boundaries.

### 4.3 Evaluation metrics and loss function

We employ two standard metrics to quantitatively evaluate the segmentation performance:

Dice Similarity Coefficient (DSC): Measures the voxel-wise overlap between the prediction P and the ground truth G, was calculated using Equation 13:

(13) $$DSC=\frac{2|P\cap G|}{\left|P|+|G\right|}$$

95% Hausdorff Distance (HD95): Measures the boundary distance. It calculates the 95th percentile of the distances between boundary points in P and G. A lower HD95 indicates better boundary delineation quality (Equation 14):

(14) $$HD95=max(h_{95}(P,G),h_{95}(G,P))$$

To improve segmentation performance and accuracy, we combine the Dice loss and the Binary Cross-Entropy (BCE) loss into a hybrid loss function.

The Dice loss is defined as Equation 15:

(15) $$L_{Dice\;}=1-\frac{2\sum_{i=1}^{N}p_{i}g_{i}+\epsilon }{\sum_{i=1}^{N}p_{i}+\sum_{i=1}^{N}g_{i}+\epsilon }$$

where *g_i_* and *p_i_* denote the ground-truth label and the model prediction, respectively, and *ϵ* is a smoothing factor to prevent division by zero.

The BCE loss is formulated as Equation 16:

(16) $$L_{BCE}=-\frac{1}{N}\sum_{i=1}^{N}\left[g_{i}log(p_{i})+(1-g_{i})log(1-p_{i})\right]$$

where *N* denotes the number of samples, *g_i_* is the ground-truth label of the sample, and *p_i_* represents the predicted positive-class probability.

The joint loss function is expressed as Equation 17:

(17) $$L_{Total\;}=wL_{Dice\;}+(1-w)\;L_{BCE}$$

where *w* is weighting coefficients that balance the contributions of the BCE and Dice terms. In this work, *w* is set to 0.5.

### 4.4 Comparative experiments

To validate the effectiveness of LiteFreqMamba, we conduct comprehensive comparisons against prevailing state-of-the-art methods on both BraTS2020 and BraTS2021 datasets. We include representative methods from three categories: CNN-based, Transformer-based, and Mamba-based. All models are evaluated with a standardized input patch size of 128×128×128 to ensure fair comparison of computational complexity. ALL models were retrained using the same patient-level split, preprocessing, augmentation, optimizer, learning rate, batch size, number of epochs, loss function, input patch size, and inference protocol as LiteFreqMamba.

**Table 1** summarizes the results on the BraTS2020 validation set. LiteFreqMamba achieves an average Dice score of 88.07%, surpassing the strong baseline SegMamba by 1.20% and SwinUNETR by 1.98%. Notably, in terms of boundary precision, our method reduces the average HD95 to 7.99 mm, significantly outperforming nnU-Net and TransBTS. The substantial improvement in the Enhancing Tumor region highlights the efficacy of our frequency-aware encoder in delineating fine, irregular boundaries even with limited training data.

**Table 1:** Quantitative comparison on the BraTS 2020 validation set.

Methods	Dice (%)↑				HD95 (mm)↓			
	WT	TC	ET	Avg.	WT	TC	ET	Avg.
nnU-Net (27)	90.82 ± 0.67	85.34 ± 0.44	79.41 ± 0.48	85.19 ± 0.45	6.82 ± 0.35	8.21 ± 0.43	19.43 ± 0.13	11.49 ± 0.59
TransBTS (15)	90.15 ± 0.19	84.82 ± 0.19	78.93 ± 0.63	84.63 ± 0.31	7.14 ± 0.65	9.05 ± 0.56	20.12 ± 0.29	12.10 ± 0.33
SwinUNETR (11)	91.53 ± 0.46	86.21 ± 0.15	80.54 ± 0.30	86.09 ± 0.55	6.12 ± 0.32	7.53 ± 0.26	17.85 ± 0.22	10.50 ± 0.40
U-Mamba (23)	91.85 ± 0.27	86.63 ± 0.21	81.22 ± 0.18	86.57 ± 0.66	5.84 ± 0.16	7.12 ± 0.41	16.51 ± 0.45	9.82 ± 0.53
SegMamba (24)	92.01 ± 0.14	87.05 ± 0.54	81.54 ± 0.60	86.87 ± 0.11	5.63 ± 0.58	6.91 ± 0.64	15.92 ± 0.36	9.49 ± 0.57
Ours	92.64 ± 0.52	88.42 ± 0.61	83.15 ± 0.38	88.07 ± 0.37	4.92 ± 0.49	5.81 ± 0.51	13.24 ± 0.25	7.99 ± 0.21

Values are presented as mean ± standard deviation. The upward arrow indicates that higher values represent better performance; the downward arrow indicates that lower values represent better performance. For Dice, a higher score means better segmentation overlap; for HD95, a lower score means better boundary alignment.

**Table 2** presents the results on the larger BraTS2021 dataset. Benefiting from the increased data scale, LiteFreqMamba achieves a new state-of-the-art Average Dice of 91.24% and an impressive HD95 of 4.49 mm. Compared to SwinUNETR, our model improves the Dice score by 1.81% and reduces the HD95 by 1.71 mm. Crucially, when compared to SegMamba, which also utilizes state space models, LiteFreqMamba achieves a 15.4% reduction in boundary error. These results suggest that explicit wavelet decomposition can improve the representation of fine boundary details in Mamba-based segmentation. Although the observed reduction in HD95 is consistent with the motivation of addressing insufficient high-frequency boundary representation, it does not by itself provide a direct measurement of spectral bias.

**Table 2:** Quantitative comparison on the BraTS 2021 validation set.

Methods	Dice (%)↑				HD95 (mm)↓			
	WT	TC	ET	Avg.	WT	TC	ET	Avg.
nnU-Net (27)	91.52 ± 0.65	88.63 ± 0.18	86.61 ± 0.35	88.92 ± 0.32	5.21 ± 0.11	6.42 ± 0.17	8.93 ± 0.49	6.85 ± 0.29
TransBTS (15)	90.84 ± 0.23	87.21 ± 0.67	87.52 ± 0.14	88.52 ± 0.50	5.53 ± 0.52	6.81 ± 0.22	9.12 ± 0.41	7.15 ± 0.30
SwinUNETR (11)	92.13 ± 0.47	89.42 ± 0.59	86.74 ± 0.66	89.43 ± 0.22	4.82 ± 0.25	5.93 ± 0.61	7.84 ± 0.46	6.20 ± 0.23
U-Mamba (23)	92.35 ± 0.53	89.81 ± 0.32	87.63 ± 0.61	89.93 ± 0.57	4.41 ± 0.63	5.22 ± 0.34	6.91 ± 0.62	5.51 ± 0.65
SegMamba (24)	92.51 ± 0.16	90.13 ± 0.24	87.82 ± 0.26	90.15 ± 0.45	4.23 ± 0.42	5.04 ± 0.12	6.65 ± 0.33	5.31 ± 0.54
Ours	93.12 ± 0.48	91.45 ± 0.27	89.14 ± 0.59	91.24 ± 0.43	3.52 ± 0.37	4.13 ± 0.31	5.82 ± 0.56	4.49 ± 0.39

Values are presented as mean ± standard deviation. The upward arrow indicates that higher values represent better performance; the downward arrow indicates that lower values represent better performance. For Dice, a higher score means better segmentation overlap; for HD95, a lower score means better boundary alignment.

**Figure 2:**
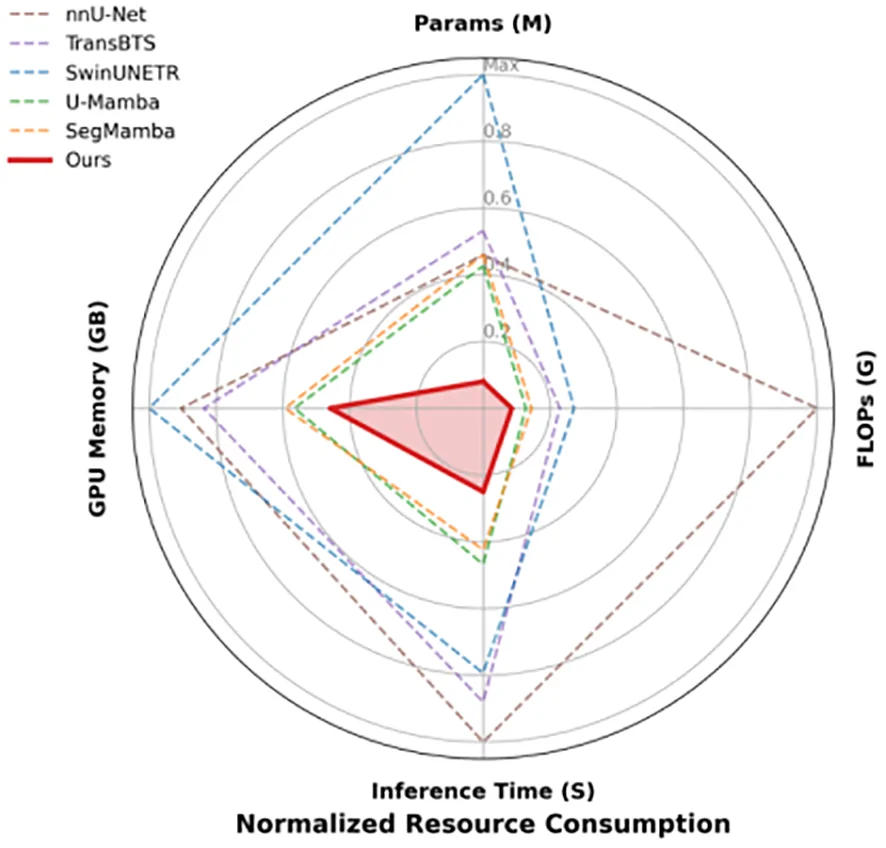
Radar plot of efficiency comparison on BraTS2021. We visualize the relative efficiency of different methods using normalized scores for Params, FLOPs, Inference Time and GPU Memory. All scores are computed under the same input patch size (128×128×128). LiteFreqMamba achieves a favorable trade-off among compactness and computational efficiency compared with baselines.

To provide a more intuitive view of efficiency trade-offs, we summarize the computational overhead of different methods in terms of parameters, FLOPs Inference Time and GPU Memory, As illustrated in **Figure 2**.

Each axis represents a normalized efficiency score converted from raw metrics. As shown, LiteFreqMamba exhibits a dominant advantage in model compactness, achieving the highest score on the Params axis with only 4.96M parameters. This corresponds to an 82.61% reduction in parameters compared with SegMamba and a 92.03% reduction compared with SwinUNETR. Despite the drastically reduced parameterization, LiteFreqMamba remains computationally efficient in terms of FLOPs, demonstrating a favorable balance between lightweight design and practical inference cost.

These results suggest that LiteFreqMamba is not only accurate but also lightweight-by-design, making it a strong candidate for deployment under constrained computational budgets.

### 4.5 Ablation experiments

To verify the effectiveness of each proposed component in LiteFreqMamba, we conduct a comprehensive ablation study on the BraTS2021 dataset. We establish a Baseline model, which is a standard U-shaped architecture employing standard 3D convolutions in the encoder and decoder, pure Mamba blocks in the bottleneck, and simple concatenation for skip connections. To disentangle the contributions of frequency-aware learning and hybrid context modeling, we evaluated the proposed modules individually. We report results for the decoder, DFS-Conv, MAHB, and FCSC when added separately to the baseline.

The independent ablation results reveal the distinct contributions of the proposed components are reported in **Table 3**. Among the individual modules, MAHB provides the largest performance gain. Adding MAHB improves the average Dice score from 89.57% to 90.32% and reduces the average HD95 from 6.12 to 5.45. The most notable improvement is observed for the TC region, whose Dice score increases by 0.99 percentage points. This result demonstrates that the sequential integration of Mamba and self-attention effectively combines long-range dependency modeling with global relational reasoning, which is particularly beneficial for anatomically complex regions.

**Table 3:** Ablation study of key components on BraTS2021 dataset.

Model	Dice (%)↑				HD95 (mm)↓			
	WT	TC	ET	Avg.	WT	TC	ET	Avg.
Baseline	91.85 ± 0.23	89.25 ± 0.12	87.62 ± 0.43	89.57 ± 0.17	5.15 ± 0.45	5.75 ± 0.21	7.45 ± 0.28	6.12 ± 0.19
Baseline + DFS	92.28 ± 0.35	89.74 ± 0.29	88.03 ± 0.52	90.02 ± 0.23	4.62 ± 0.13	5.12 ± 0.53	6.68 ± 0.16	5.47 ± 0.23
Baseline + FCSC	92.07 ± 0.56	89.57 ± 0.41	87.96 ± 0.59	89.87 ± 0.30	4.77 ± 0.65	5.31 ± 0.58	6.86 ± 0.33	5.65 ± 0.31
Baseline + MAHB	92.42 ± 0.63	90.24 ± 0.57	88.31 ± 0.24	90.32 ± 0.29	4.63 ± 0.17	5.03 ± 0.49	6.69 ± 0.26	5.45 ± 0.21
Baseline + LWT	91.81 ± 0.61	89.21 ± 0.44	87.51 ± 0.39	89.51 ± 0.28	5.21 ± 0.66	5.81 ± 0.62	7.51 ± 0.47	6.18 ± 0.34
Ours	93.12 ± 0.48	91.45 ± 0.27	89.14 ± 0.59	91.24 ± 0.43	3.52 ± 0.37	4.13 ± 0.31	5.82 ± 0.56	4.49 ± 0.39

Values are presented as mean ± standard deviation. The upward arrow indicates that higher values represent better performance; the downward arrow indicates that lower values represent better performance. For Dice, a higher score means better segmentation overlap; for HD95, a lower score means better boundary alignment.

DFS-Conv yields the second most substantial improvement, increasing the average Dice score to 90.02% and reducing the average HD95 to 5.47. Its impact is especially evident in boundary localization, with the ET HD95 decreasing from 7.45 to 6.68. This behavior is consistent with its wavelet-based high-frequency decomposition, which explicitly enhances fine-grained boundary and structural cues.

FCSC also produces consistent improvements, raising the average Dice score to 89.87% and reducing the average HD95 to 5.65. Although its standalone gain is smaller than those of DFS-Conv and MAHB, FCSC improves all three tumor regions. This suggests that SE-based calibration can effectively select and reinject informative encoder features. Its relatively modest independent contribution is expected because, without DFS-Conv, the encoder provides fewer explicitly enhanced high-frequency features for recalibration.

By contrast, LWT-3D Conv slightly reduces the average Dice score to 89.51% and increases the average HD95 to 6.18. This indicates a minor accuracy-efficiency trade-off when it is used alone. Nevertheless, its role in the complete architecture remains important because the enhanced features produced by DFS-Conv and FCSC can compensate for potential reconstruction losses.

The complete model achieves 91.24% average Dice and 4.49 average HD95, confirming strong complementary interactions among the modules rather than simple additive effects.

### 4.6 Qualitative results

**Figure 3:**
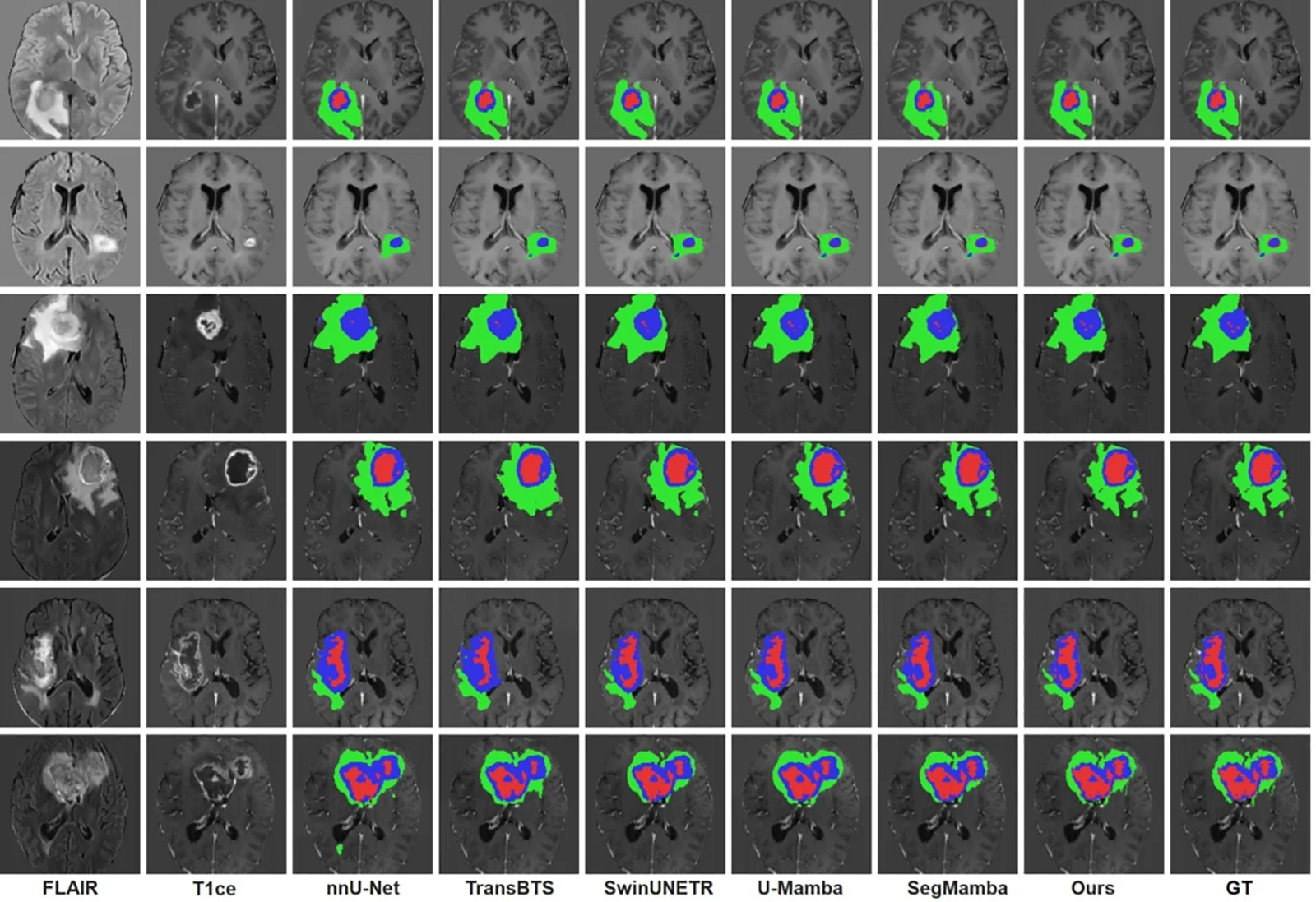
Visual comparison of segmentation results on representative BraTS2021 validation cases. Columns from left to right: T1ce input, Ground Truth, nnU-Net, SwinUNETR, SegMamba, and Ours. Enhancing Tumor (ET), Edema (ED), and Tumor Core (TC) are visualized in red, green, and blue, respectively. LiteFreqMamba produces more anatomically consistent segmentation masks, particularly in challenging cases involving small lesions, multi-focal tumor cores, and heterogeneous tumor boundaries.

To qualitatively evaluate the segmentation capability of LiteFreqMamba, we compare the visual segmentation results of different methods on representative BraTS2021 validation cases, as shown in **Figure 3**. The selected examples include both relatively easy cases and highly challenging scenarios involving multi-focal tumor cores, minute enhancing tumor regions, large-volume lesions, and heterogeneous tumor structures. These cases are intentionally chosen to assess the robustness of different architectures under diverse tumor distributions and boundary conditions.

For relatively regular medium-sized tumors, all methods are able to recover the major tumor regions with reasonable accuracy. Nevertheless, subtle differences can still be observed around the boundaries of the enhancing tumor. In particular, nnU-Net and SwinUNETR exhibit slight under-segmentation near the ET margins, resulting in contours that contract inward relative to the ground truth. By contrast, LiteFreqMamba produces boundaries that more closely align with the ground truth and better preserve local structural continuity.

The superiority of LiteFreqMamba becomes more evident in challenging cases. In the multi-focal tumor core example, several competing methods either partially miss the satellite lesion or underestimate its spatial extent. LiteFreqMamba, however, preserves both the morphology and spatial continuity of the satellite focus more faithfully. Similarly, in cases containing multiple minute ET regions, the proposed method successfully detects all weak lesions while maintaining more complete lesion shapes. In comparison, competing methods tend to either miss small targets or produce fragmented predictions with noticeable under-segmentation.

For large-volume and highly heterogeneous tumors, LiteFreqMamba demonstrates robustness in preserving global anatomical structure. Compared with CNN- and Transformer-based baselines, the proposed method generates more spatially coherent segmentation masks with improved boundary consistency and fewer discontinuous regions. In addition, the predicted edema regions remain anatomically plausible and better aligned with the surrounding tumor structures.

Overall, the qualitative comparisons indicate that LiteFreqMamba achieves more reliable delineation across a wide range of tumor patterns, including weak ET regions, multi-focal lesions, and large heterogeneous tumors. These observations are consistent with the quantitative improvements reported in **Table 1** and **2**, particularly in boundary-sensitive metrics such as HD95.

### 4.7 Error analysis and boundary visualization

**Figure 4:**
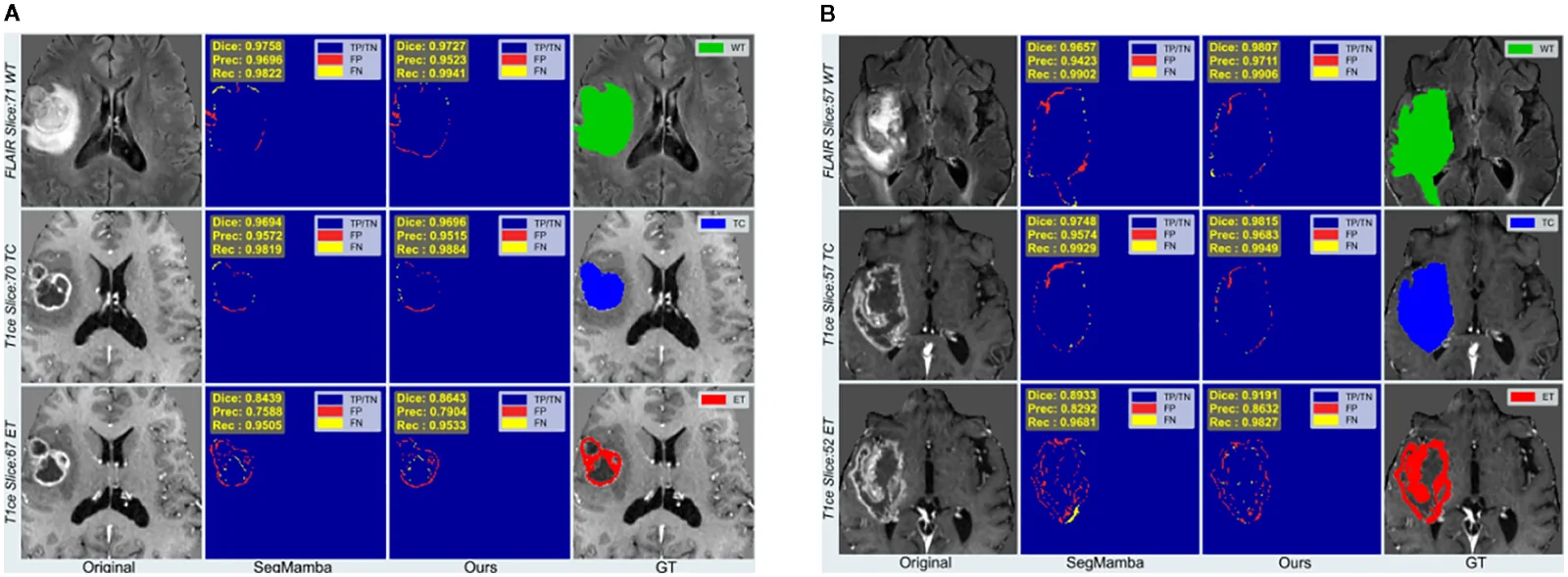
Error-map based comparison between SegMamba and LiteFreqMamba on challenging BraTS 2020 cases. FP (false positives) are shown in red, FN (false negatives) are shown in blue. **(a)** Multi-focal tumor core with edema-dominant appearance. **(b)** Large-volume tumor case. LiteFreqMamba produces fewer FN and improves boundary completeness and continuity.

To more clearly demonstrate the boundary refinement and error suppression capability of LiteFreqMamba, we provide error-map visualizations in **Figure 4**, where false positives (FP) are highlighted in red and false negatives (FN) in blue. **Figure 4a** corresponds to a challenging case with multi-focal tumor cores and edema-dominant appearance, where the enhancing tumor (ET) is relatively small; **Figure 4b** shows a large-volume tumor case that tests the global structural consistency of the segmentation.

In **Figure 4a**, our method better preserves the satellite lesion, whereas SegMamba still exhibits partial under-segmentation. the edema-dominant background and small ET regions make the segmentation particularly sensitive to noise and class confusion. Compared with SegMamba, LiteFreqMamba produces noticeably fewer false negatives around the small enhancing tumor, indicating improved sensitivity to weak ET signals. Meanwhile, the false positives in edema-adjacent regions are reduced, suggesting that the proposed frequency-calibrated skip fusion effectively suppresses task-irrelevant high-frequency responses that may otherwise be mistaken as tumor boundaries.

In **Figure 4b**, the tumor occupies a large spatial extent with complex peripheral boundaries. In this scenario, segmentation errors often manifest as fragmented contours or incomplete coverage near the tumor margins. The error maps show that LiteFreqMamba achieves better boundary continuity, with reduced false negatives along the peripheral tumor region and fewer spurious false positives in surrounding tissues. This indicates that the proposed hybrid global-local modeling improves volumetric coherence for large structures while preserving fine boundary details.

Overall, the error-map analysis provides intuitive evidence that LiteFreqMamba improves not only volumetric overlap but also boundary precision in both edema-dominant multi-focal cases and large-volume tumors, which is consistent with the gains in boundary-sensitive metrics such as HD95 reported in **Table 1** and **2**.

### 4.8 Grad-CAM visualization

**Figure 5:**
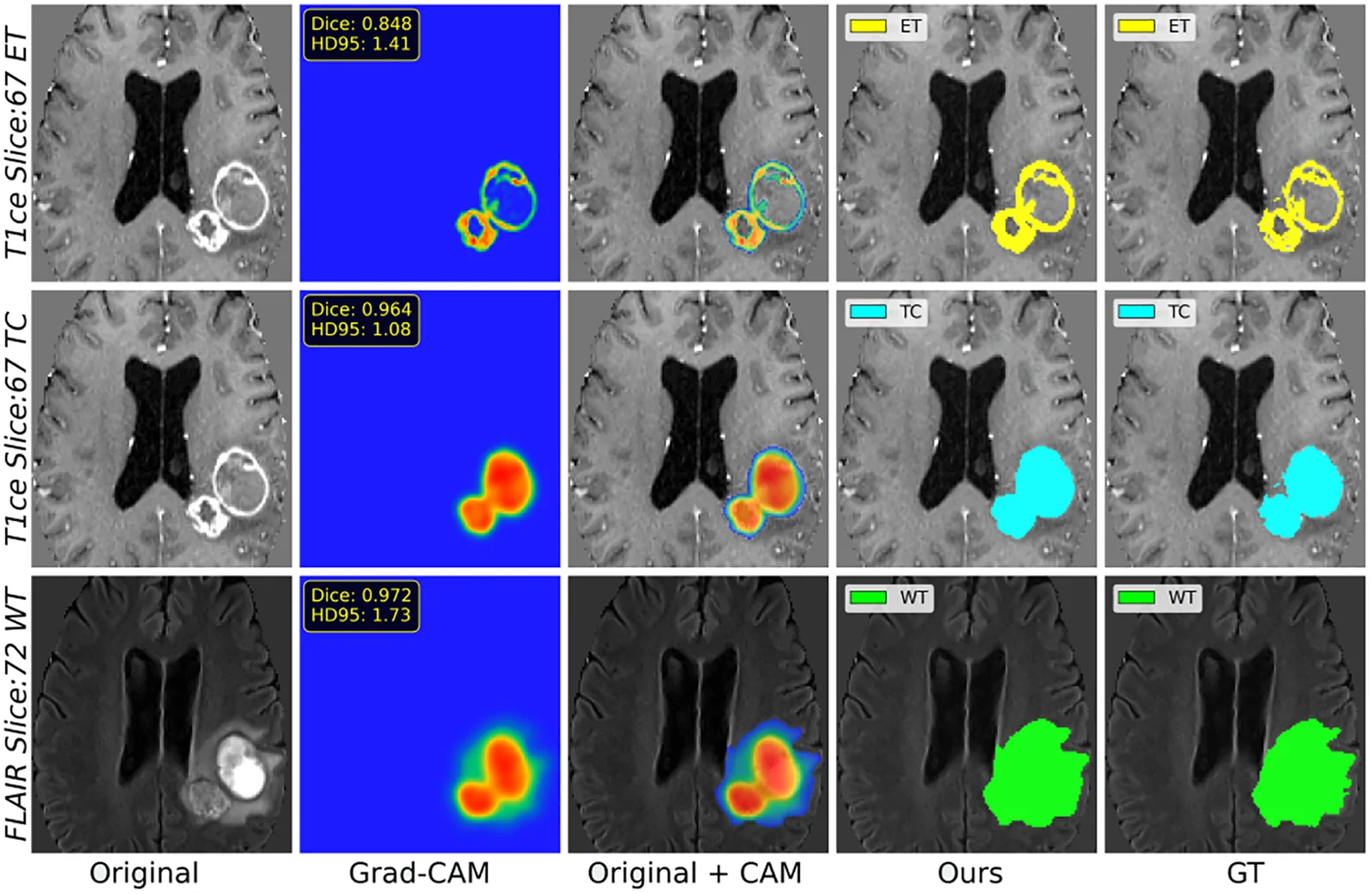
Grad-CAM visualization of LiteFreqMamba. Three rows correspond to ET, TC, and WT, respectively. Columns from left to right (1): raw modality slice (2), Grad-CAM activation map (3), activation overlay on the raw slice (4), Ours prediction overlay (ET: yellow, TC: light blue, WT: green), and (5) GT overlay (ET: yellow, TC: light blue, WT: green). The proposed framework generates activation responses that are spatially concentrated around clinically relevant tumor regions and highly consistent with the corresponding segmentation masks.

To further investigate the internal decision-making behavior of LiteFreqMamba, we employ Grad-CAM to visualize class-specific activation responses generated by the proposed network.The visualization results are shown in **Figure 5**, where activation maps are presented for the three clinically important tumor regions: Enhancing Tumor, Tumor Core, and Whole Tumor.

As illustrated in **Figure 5**, the visualization is organized into three rows corresponding to ET, TC, and WT, respectively. For each row, the first column shows the original grayscale MRI slice used as input. The second column presents the corresponding Grad-CAM activation map, highlighting the spatial regions that contribute most strongly to the prediction of the target class. The third column overlays the activation map on the original image to facilitate anatomical interpretation. The fourth and fifth columns show the predicted segmentation and the corresponding ground truth, respectively, overlaid on the same grayscale slice for direct visual comparison. For consistency, ET, TC, and WT are visualized in yellow, light blue, and green, respectively.

The Grad-CAM results reveal several important observations. First, the activations are spatially concentrated around regions associated with the predicted tumor classes. These visualizations provide qualitative evidence that the model attends to image regions relevant to the segmentation output. Second, the activation responses progressively expand from ET to TC and further to WT, reflecting the hierarchical relationship among the three tumor subregions. In particular, the ET activations are highly localized around small enhancing lesions, while the WT activations capture broader edema-related structures. This behavior suggests that the proposed frequency-aware encoder and hybrid bottleneck are capable of simultaneously preserving local boundary-sensitive information and global contextual structures.

Moreover, the activation overlays exhibit strong spatial consistency with both the predicted segmentation and the GT masks. The highlighted regions align well with tumor boundaries and lesion extents, especially in complex regions containing heterogeneous tumor appearance. These observations provide qualitative evidence that LiteFreqMamba focuses on clinically relevant structures and avoids excessive activation in surrounding normal tissues, thereby contributing to the improved boundary delineation and reduced false positives observed in previous experiments.

### 4.9 Multi-view visualization

**Figure 6:**
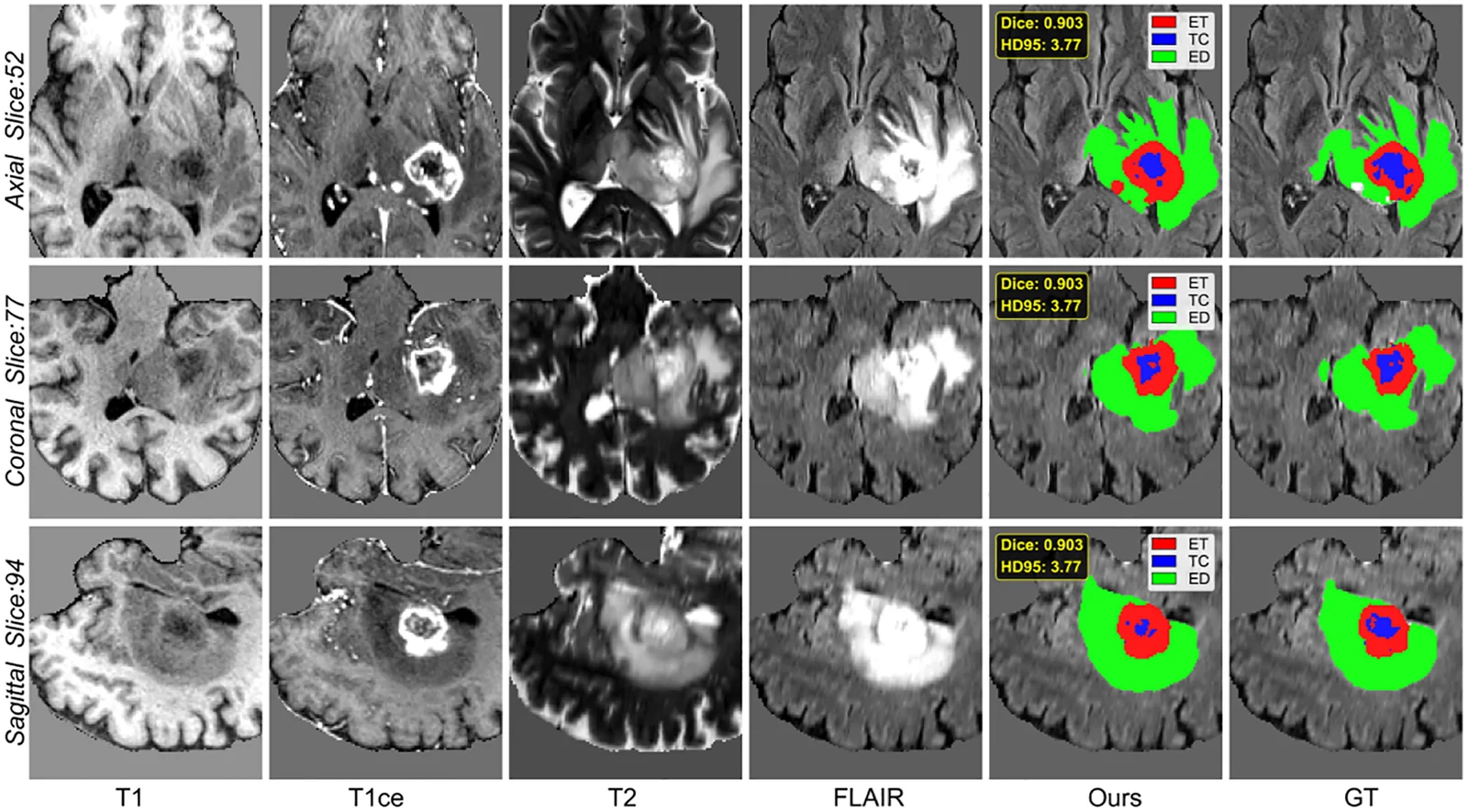
Multi-view visualization of segmentation results on axial, coronal, and sagittal planes. Rows from top to bottom: axial, coronal, sagittal. Columns from left to right: T1, T1ce, T2, FLAIR, Ours overlay, and GT overlay. In the overlay columns, ET, TC and ED are shown in red, blue and green, respectively. The proposed framework produces spatially coherent and anatomically consistent tumor delineation across different anatomical planes.

To further evaluate the volumetric consistency of LiteFreqMamba, we visualize the segmentation results from three orthogonal anatomical views, namely the axial, coronal, and sagittal planes. The corresponding results are presented in **Figure 6**, where each row represents one anatomical plane. For every view, the first four columns show the four MRI modalities (T1, T1ce, T2, and FLAIR), followed by the segmentation overlays of Ours and the corresponding ground truth on the same slice. In the overlay visualizations, the three tumor subregions are simultaneously displayed, where Enhancing Tumor, Tumor Core, and Edema are colored in red, blue, and green, respectively.

The multi-view visualization reveals that LiteFreqMamba maintains strong structural consistency across different anatomical orientations. Unlike slice-wise methods that may produce fragmented or discontinuous predictions between adjacent slices, the proposed framework generates spatially coherent tumor regions in all three planes. In particular, the ET and TC regions remain anatomically stable when observed from different viewing directions, indicating that the proposed hybrid state-space modeling effectively preserves volumetric continuity.

Moreover, the multi-modal visualization highlights the complementary contribution of different MRI modalities. T1ce provides clear enhancement information for ET regions, while FLAIR and T2 contribute stronger responses for edema-related structures. The segmentation overlays demonstrate that LiteFreqMamba is capable of integrating these heterogeneous modality-specific cues into a unified volumetric representation. As a result, the predicted tumor regions exhibit strong agreement with the GT not only in terms of global shape, but also in local structural details such as boundary continuity and lesion extent.

Overall, the multi-view analysis provides additional qualitative evidence that LiteFreqMamba achieves anatomically plausible and volumetrically consistent segmentation across different spatial orientations, further supporting the effectiveness of the proposed frequency-aware visual state-space framework.

## 5 Discussion

The experimental results presented in Sections 4.4-4.9 consistently demonstrate that the proposed design achieves a favorable balance between segmentation accuracy, boundary precision, and computational efficiency. In particular, the quantitative comparisons, qualitative visualizations, and error-map analyses collectively validate the effectiveness of explicitly incorporating frequency-aware modeling into lightweight visual state-space architectures for volumetric brain tumor segmentation.

A key observation of this study is the importance of mitigating the spectral bias inherent in state-space based segmentation models. Although recent Mamba-based methods such as SegMamba already provide strong long-range dependency modeling, they still exhibit limitations in preserving high-frequency tumor boundaries, especially in weak and fragmented ET regions. This behavior is reflected in the comparative experiments, where standard Mamba-based approaches achieve competitive Dice scores but remain inferior in boundary-sensitive metrics such as HD95. The proposed DFS-Conv encoder effectively alleviates this issue by explicitly decomposing volumetric features into low- and high-frequency components. The substantial reduction in ET HD95 observed in **Tables 1**, **2** confirms the effectiveness of this frequency-aware design.

The ablation experiments in Section 4.5 also reveal that lightweight design and strong representation capability are not mutually exclusive. Replacing the conventional decoder with the proposed LWT-3D Conv blocks significantly reduces parameter count and FLOPs while causing only negligible performance degradation. This observation is particularly important for practical deployment, as volumetric medical segmentation models are often constrained by GPU memory and inference latency. Building upon this lightweight baseline, the introduction of DFS-Conv, the Hybrid Bottleneck, and FCSC yields progressively improved segmentation performance, indicating that each proposed module contributes synergistically to the final framework.

Another important finding is that efficient global modeling alone is insufficient for robust 3D segmentation. While SS2D-based visual state-space scanning effectively captures long-range spatial dependencies, it may still overlook dense local interactions and precise structural continuity. The proposed MAHB addresses this limitation by combining efficient state-space modeling with lightweight self-attention. The resulting dual-view design simultaneously captures global structural context and local anatomical details, leading to improved WT and TC segmentation performance. Compared with Transformer-based architectures such as SwinUNETR, LiteFreqMamba achieves similar or superior contextual modeling with substantially lower computational overhead.

The visualization results in Sections 4.6-4.9 provide additional insight into the behavior of the proposed framework. In the qualitative comparisons of Section 4.6, LiteFreqMamba demonstrates robustness across diverse tumor patterns, including multi-focal lesions, minute ET regions, and large heterogeneous tumors. The error-map analysis in **Figure 4** further highlights the importance of effective feature calibration. In the edema-dominant multi-focal case shown in **Figure 4a**, competing methods exhibit noticeable false negatives around small ET regions and false positives near edema boundaries. Similarly, in the large-volume tumor case shown in **Figure 4b**, baseline methods tend to produce fragmented boundaries and incomplete tumor coverage. By contrast, LiteFreqMamba generates more spatially coherent predictions with reduced false positives and false negatives, indicating that the proposed FCSC effectively suppress noisy high-frequency responses while preserving informative boundary cues.

The Grad-CAM visualizations and multi-view segmentation results further strengthen the interpretability of the proposed framework. The Grad-CAM activation maps demonstrate that LiteFreqMamba consistently focuses on clinically relevant tumor regions rather than background structures, while the axial, coronal, and sagittal visualizations reveal strong volumetric consistency across different anatomical planes. These observations suggest that the proposed frequency-aware state-space design not only improves quantitative metrics but also enhances the anatomical plausibility of the resulting segmentation maps.

Despite these encouraging results, several limitations remain. First, the current framework assumes the availability of all MRI modalities during inference, whereas missing modalities are common in real-world clinical settings. Future work will investigate modality-agnostic or cross-modal learning strategies to improve robustness under incomplete inputs. Second, although the proposed (2 + 1)D frequency decomposition significantly improves efficiency, these segmentation results do not directly measure the frequency response of the learned representations and therefore cannot conclusively establish that the spectral bias of Mamba-based models has been mitigated. A direct assessment would require additional frequency-domain analyses. Finally, the current evaluation is limited to brain tumor datasets, and future studies will explore the applicability of LiteFreqMamba to other volumetric segmentation tasks, such as liver, kidney, and pelvic organ segmentation.

## 6 Conclusion and future work

This work introduces LiteFreqMamba, a lightweight frequency-spatial visual state-space framework for 3D brain tumor segmentation. The proposed architecture is designed to improve high-frequency boundary representation and spatial dependency modeling while maintaining computational efficiency. DFS-Conv uses (2 + 1)D wavelet-based decomposition to preserve boundary-related features in shallow stages, MAHB combines SS2D-based state-space modeling with lightweight self-attention to capture global and dense local dependencies, and FCSC calibrates skip features to suppress task-irrelevant responses during encoder–decoder fusion.

Experiments on BraTS2020 and BraTS2021 show competitive segmentation performance, with average Dice scores of 88.07% and 91.24% and HD95 values of 7.99 and 4.49 mm, respectively. The model contains 4.96M parameters and 123.98 GFLOPs, using 82.61% fewer parameters than SegMamba and 92.03% fewer than SwinUNETR. The ablation and boundary-sensitive results support the effectiveness of the proposed frequency-aware design for improving tumor boundary delineation and maintaining a favorable accuracy–efficiency trade-off.

Nevertheless, the current experiments do not directly measure the frequency response of the learned representations and therefore do not provide conclusive evidence that the spectral bias of Mamba-based models has been mitigated. In addition, the evaluation is limited to the BraTS2020 and BraTS2021 benchmarks. Future work will investigate frequency-response analysis, frequency-specific ablations, external multicenter validation, missing-modality robustness, and the practical deployment efficiency of the proposed framework.

## Data Availability

Publicly available datasets were analyzed in this study. This data can be found here: The datasets used in this study is publicly available from Multimodal Brain Tumor Segmentation Challenge. BraTS2020: https://www.med.upenn.edu/cbica/brats2020/data.html; BraTS2021: https://www.kaggle.com/datasets/dschettler8845/brats-2021-task1. All experiments were conducted solely on this dataset and strictly adhered to the competition’s data usage terms.
